# Damage-associated molecular patterns and fibrinolysis perturbation are associated with lethal outcomes in traumatic injury

**DOI:** 10.1186/s12959-023-00536-w

**Published:** 2023-09-06

**Authors:** Kenshin Shimono, Takashi Ito, Chinatsu Kamikokuryo, Shuhei Niiyama, Shingo Yamada, Hirokazu Onishi, Hideaki Yoshihara, Ikuro Maruyama, Yasuyuki Kakihana

**Affiliations:** 1 Department of Emergency and Intensive Care Medicine, Kagoshima University Graduate School of Medical and Dental Sciences, Kagoshima, Japan; 2 Department of Biomedical Laboratory Sciences, Faculty of Life Sciences, Kumamoto University, 4-24-1 Kuhonji, Kumamoto, 862-0976 Japan; 3 Shino-Test Corporation, R&D Center, Sagamihara, Japan; 4 Emergency and Critical Care Center, Kagoshima City Hospital, Kagoshima, Japan; 5 Department of Systems Biology in Thromboregulation, Kagoshima University Graduate School of Medical and Dental Sciences, Kagoshima, Japan

**Keywords:** Damage-associated molecular patterns (DAMPs), Trauma-induced coagulopathy, Histone H3, Plasmin-α2-PI complex (PIC), Plasminogen activator inhibitor-1 (PAI-1)

## Abstract

**Background:**

Upon cellular injury, damage-associated molecular patterns (DAMPs) are released into the extracellular space and evoke proinflammatory and prothrombotic responses in animal models of sterile inflammation. However, in clinical settings, the dynamics of DAMP levels after trauma and links between DAMPs and trauma-associated coagulopathy remain largely undetermined.

**Methods:**

Thirty-one patients with severe trauma, who were transferred to Kagoshima City Hospital between June 2018 and December 2019, were consecutively enrolled in this study. Blood samples were taken at the time of delivery, and 6 and 12 h after the injury, and once daily thereafter. The time-dependent changes of coagulation/fibrinolysis markers, including thrombin-antithrombin complex, α2-plasmin inhibitor (α2-PI), plasmin-α2-PI complex, and plasminogen activator inhibitor-1 (PAI-1), and DAMPs, including high mobility group box 1 and histone H3, were analyzed. The relationship between coagulation/fibrinolysis markers, DAMPs, Injury Severity Score, in-hospital death, and amount of blood transfusion were analyzed.

**Results:**

The activation of coagulation/fibrinolysis pathways was evident at the time of delivery. In contrast, PAI-1 levels remained low at the time of delivery, and then were elevated at 6–12 h after traumatic injury. Histone H3 and high mobility group box 1 levels were elevated at admission, and gradually subsided over time. PAI-1 levels at 6 h were associated with serum histone H3 levels at admission. Increased histone H3 levels and plasmin-α2-PI complex levels were associated with in-hospital mortality. α2-PI levels at admission showed the strongest negative correlation with the amount of blood transfusion.

**Conclusion:**

The elevation of histone H3 levels and fibrinolysis perturbation are associated with fatal outcomes in patients with traumatic injury. Patients with low α2-PI levels at admission tend to require blood transfusion.

## Background

Mortality from traumatic injury remains a global health issue, leading to over four million deaths a year [1]. The burden is highest in individuals younger than 50 years of age, among whom traumatic injury is the second cause of death [2]. Notably, uncontrolled bleeding is the most common preventable cause of death for patients with severe injury [3]. Coagulopathy associated with severe injury complicates the control of bleeding and is associated with increased morbidity and mortality in trauma patients [4]. Consequently, there is intense interest worldwide in the pathogenesis of trauma-induced coagulopathy (TIC) [2].

The pathogenesis of TIC is complicated. At the beginning of trauma, the exposure of blood to the subendothelial matrix, including collagen and tissue factor, drives the localized activation of platelets and coagulation factors [3, 5]. This process is essential for effective hemostasis, and if insufficient, persistent bleeding results in massive blood loss. The persistent and/or widespread activation of platelets and coagulation factors exacerbates the loss of hemostatic factors, leading to so-called consumption coagulopathy [5]. Replacement with crystalloid, colloid, or red cell transfusion also decreases the concentration of hemostatic factors, resulting in dilutional coagulopathy [6]. The triad of hypoxia, acidosis, and hypothermia predisposes patients to further bleeding [3]. Hypoxia and acidosis impair the functional ability of platelets and coagulation factors [3]. Hemostatic defects are most evident once the body temperature falls below 33°C [7]. Shock is also associated with the development of TIC [8, 9].

Fibrinolysis is another independent risk factor for TIC [5]. Clinical data have suggested that the degree of fibrinolysis is correlated with transfusion requirement [10] and mortality in trauma patients [11]. This is related to the high levels of free tissue-type plasminogen activator (tPA) found early after trauma [9], resulting in increased plasmin generation and fibrin degradation [9, 11]. Whereas hyperfibrinolysis is a major event in early TIC, trauma-induced fibrinolytic shutdown has also been reported [12]. There is a U-shaped distribution of mortality related to fibrinolysis in response to major trauma [13]. Hyperfibrinolysis is associated with early death from exsanguination, and hypofibrinolysis is associated with delayed death due to organ failure [13]. Mechanistically, shock-induced tPA release at an early stage of trauma is associated with hyperfibrinolysis, and the induction of plasminogen activator inhibitor-1 (PAI-1) expression at a later stage of trauma is associated with fibrinolytic shutdown [14].

Traumatic injury may cause the local and systemic release of damage-associated molecular patterns (DAMPs) [15], which are endogenous molecules normally found inside cells unless released by damage. Under conditions of cellular stress or injury, DAMPs can be released into the extracellular space, activating innate immune cells [16]. Nuclear proteins including high mobility group box 1 (HMGB1) and histones, purine metabolites including ATP and uric acid, and mitochondrial components including formyl peptides and mitochondrial DNA are prototypical DAMPs [17, 18]. Extracellular HMGB1 activates monocytes to induce the surface expression of tissue factor [19], an important trigger of the extrinsic coagulation pathway. Extracellular HMGB1 also activates neutrophils to induce the extrusion of neutrophil extracellular traps [20]. Extracellular histones can aggregate platelets, leading to microvascular thrombosis and thrombocytopenia [21, 22]. Thus, growing evidence suggests that DAMPs may play an important role in the pathogenesis of coagulopathy in animal models and cellular experiments; however, the dynamics of DAMP levels after trauma and links between DAMPs and trauma-associated coagulation/fibrinolysis abnormalities remain largely undetermined. In this study, we investigated these issues by analyzing the consecutive data of severe trauma patients.

## Methods

### Study population

This was a prospective, single-center, observational study. Between June 2018 and December 2019, written informed consent was obtained from 31 severe trauma patients, who were transferred to Kagoshima City Hospital. Blood sampling was performed at the time of delivery, 6 and 12 h after injury, and once daily thereafter until the patients were transferred to the general ward. Plasma and serum samples from the patients were anonymized and used for the measurements of coagulation and fibrinolysis markers, histone H3 and HMGB1. Clinical data, including the Injury Severity Score (ISS), in-hospital death, and amounts of transfusion, were anonymized and analyzed. Severe trauma was defined as ISS ≥ 16 points. The study was compliant with the Declaration of Helsinki and approved by the Ethics Committee of Kagoshima University Graduate School of Medical and Dental Sciences, and Kagoshima City Hospital.

### Measurement of coagulation and fibrinolysis markers

The reagents used in this study were as follows: PT, HemosIL RecombiPlasTin (Instrumentation Laboratory Company, Bedford, MA, USA); APTT, HemosIL Synth ASil (Instrumentation Laboratory Company); fibrinogen, Thrombocheck Fib (Sysmex, Kobe, Japan); thrombin-antithrombin (TAT) complex, STACIA CLEIA TAT (LSI Medience, Tokyo, Japan); plasmin-alpha2-plasmininhibitor-complex (PIC), LPIA ACE PPI II (LSI Medience); PAI-1, LPIA-tPAI test (LSI Medience); α2-plasmin inhibitor (α2-PI), TESTTEAMS APL (SEKISUI MEDICAL Co., Ltd.). PT, APTT, fibrinogen, TAT, PIC, and PAI-1 were measured using an automated analyzer (STACIA, LSI Medience). The platelet count was determined using an XN-1000 automated counting device (Sysmex).

### Measurement of serum HMGB1 and histone H3 Levels

Serum HMGB1 levels were measured using an HMGB1 ELISA Kit (Shino-Test Corporation, Tokyo, Japan) according to the manufacturer’s instructions. Serum histone H3 levels were also measured using an enzyme-linked immunosorbent assay (ELISA) at Shino-Test Corporation as described previously [23].

### Statistical analyses

The relationships between coagulation and fibrinolysis markers, histone H3, and HMGB1 levels were analyzed by Spearman’s rank correlation test. A *P*-value < 0.05 was considered statistically significant. All statistical analyses were performed with EZR version 1.40 (Saitama Medical Center, Jichi Medical University, Saitama, Japan), which is a graphical user interface for R (The R Foundation for Statistical Computing, Vienna, Austria) [24]. Receiver operating characteristics curve analysis with area under the curve (AUC) was used to quantify the predictive performance of coagulation and fibrinolysis markers, HMGB1, histone H3, and ISS for hospital death.

## Results

### Baseline characteristics of the patients

The baseline characteristics of the 31 patients are shown in Table 1. All 31 patients (20 men and 11 women) had an ISS ≥ 16 points. The age of patients ranged from 24 to 91 years with a median of 78. There were 11 in-hospital deaths, which include seven deaths due to traumatic brain injuries, two deaths due to sepsis, one death due to lung contusion, and one death due to non-infectious multiple organ failure. Death due to hemorrhagic shock was absent in this study.

**Table 1:** Basic characteristics of the study patients

	**All**	**Survivors**	**Non-survivors**	*P*
	***n* = 31**	***n* = 20**	***n* = 11**	
Age (years)	78 (24–91)	76.5 (24–87)	80 (70–91)	0.027
Male sex	20 (64.5)	15 (48.4)	5 (16.1)	0.26
Injury Severity Score	30 (16–54)	30 (16–54)	38 (16–50)	0.59
SOFA score	5 (0–12)	5 (0–8)	6 (4–12)	0.11
JAAM DIC score	4 (2–6)	4 (2–6)	4 (3–5)	0.52
Lactate (mmol/L)	2.4 (1.0–8.1)	1.95 (1.0–8.1)	2.9 (1.3–6.2)	0.17
Pre-hospital Fluids (ml)	500 (0–5000)	500 (0–5000)	500 (0–1000)	0.35
Time from injury to delivery (min)	56 (19–270)	57 (24–270)	55 (19–150)	0.48
Use of tranexamic acid	22 (71.0)	13 (41.9)	9 (29.0)	0.23

Data presented as median (minimum–maximum) or *n* (%)

Pre-hospital fluids shown are approximate

Differences between survivors and non-survivors were analyzed by the Mann–Whitney U-test

### Dynamic changes in coagulation markers and fibrinolysis markers during the post-traumatic period

**Fig. 1:**
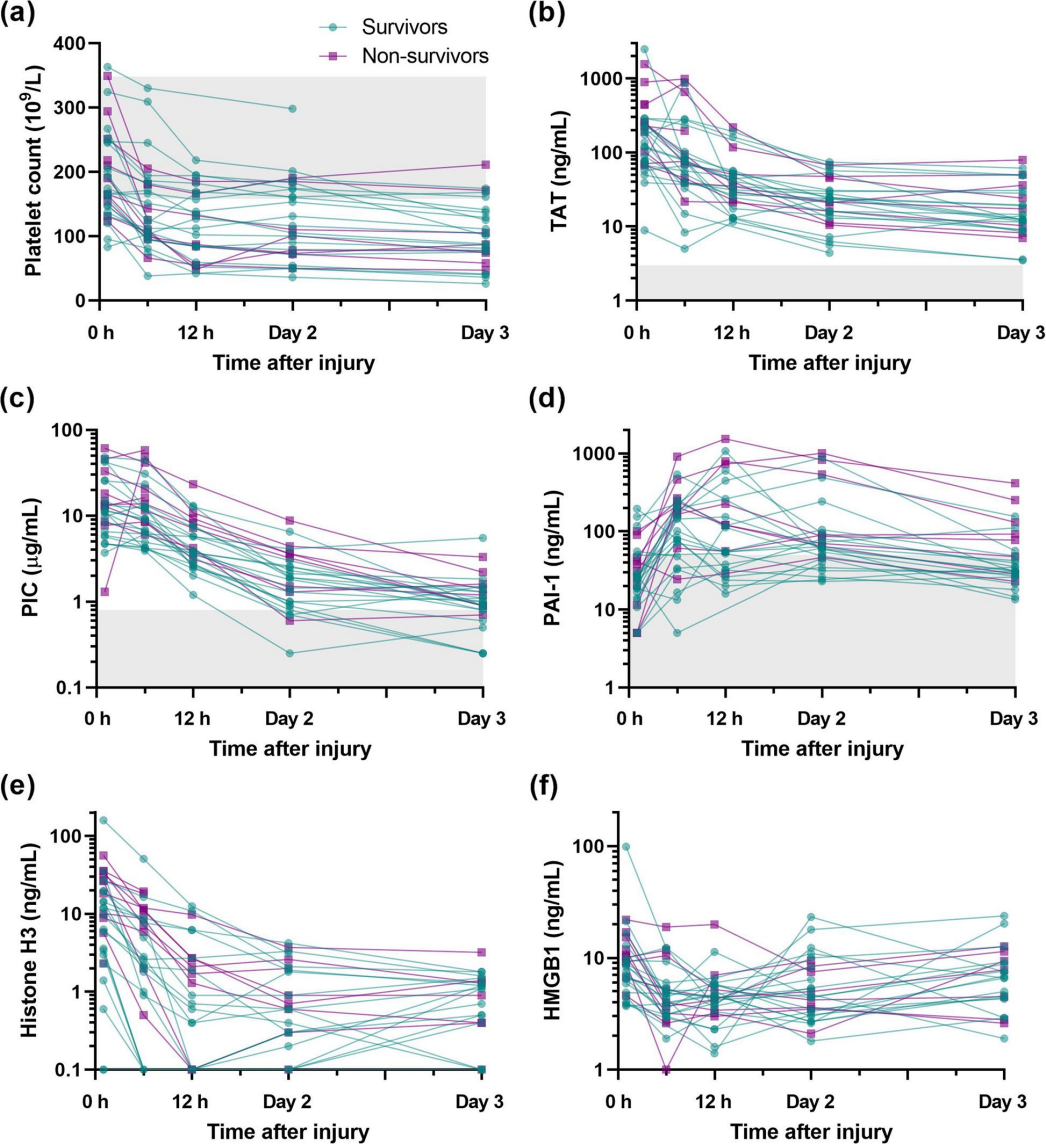
Dynamic changes in coagulation markers, fibrinolysis markers, and damage-associated molecular patterns during the post-traumatic period. Platelet counts (**a**), thrombin-antithrombin complex (TAT) levels (**b**), plasmin-alpha2-plasmin inhibitor complex (PIC) levels (**c**), plasminogen activator inhibitor-1 (PAI-1) levels (**d**), histone H3 levels (**e**), and high mobility group box 1 protein (HMGB1) levels (**f**) in patients with severe trauma at the time of delivery, 6 and 12 h after the injury, and day 2 and day 3 are shown. The gray zones indicate the reference intervals. Green and purple symbols indicate survivors and non-survivors, respectively

In our study, blood samples were taken at the time of delivery, 6 and 12 h after the injury, and once daily thereafter until the patients were transferred to the general ward. As shown in Fig. 1, platelet counts were not decreased at the time of arrival in most patients, and these subsequently decreased over time. TAT, a marker of coagulation activation, was markedly elevated at the time of arrival and gradually subsided over time. PIC, a marker of fibrinolysis activation, also showed a marked increase at the time of arrival, followed by a gradual decrease. These findings suggest that the coagulation and fibrinolysis pathways are activated immediately after a traumatic injury. In contrast, PAI-1, an inhibitor of fibrinolysis systems, remained low at the time of arrival, and then showed a marked increase at 6–12 h after traumatic injury.

### Serum histone H3 levels are increased immediately after traumatic injury and are associated with subsequent elevation of PAI-1

**Fig. 2:**
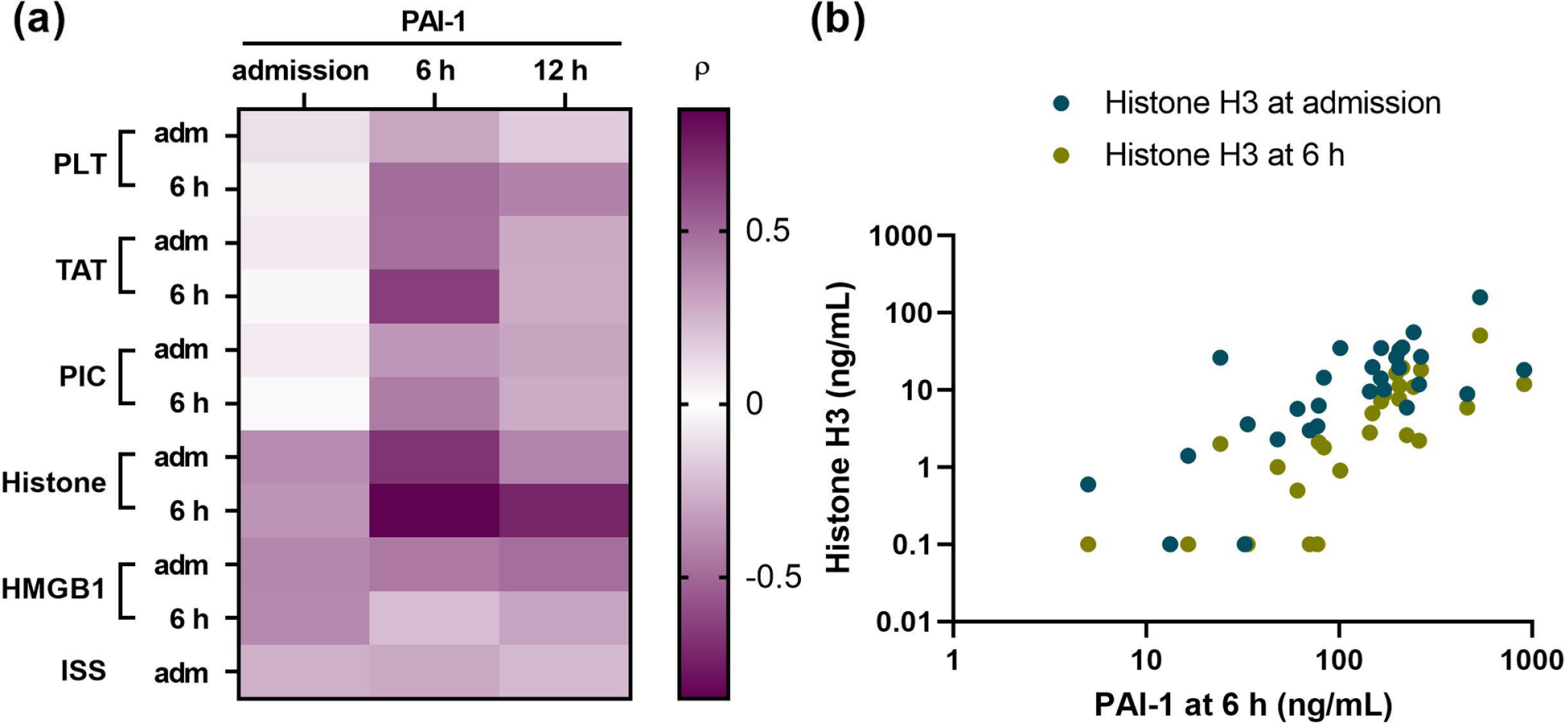
The elevation of serum histone H3 levels is associated with the subsequent elevation of plasminogen activator inhibitor-1. **a** Relationships between plasminogen activator inhibitor-1 (PAI-1) levels and platelet (PLT) counts, thrombin-antithrombin complex (TAT), plasmin-alpha2-plasmin inhibitor complex (PIC), histone H3, high mobility group box 1 protein (HMGB1), and injury severity score (ISS) levels in patients with severe trauma are shown. The intensities of the correlations are color-coded according to Spearman’s rho values, with deep purple colors indicating strong correlations. **b** Correlation between PAI-1 levels at 6 h and circulating histone H3 levels at admission (*n* = 29) and at 6 h (*n* = 29) are shown

Histone H3, a marker and mediator of cellular damage, was elevated at the time of arrival and gradually subsided over time. HMGB1, another DAMP molecule, was also elevated at the time of arrival and then decreased. In some patients, HMGB1 levels gradually increased again on days 2 and 3. Thus, most markers reached a peak at the time of arrival, although PAI-1 was an exception. We then analyzed what caused the elevation of PAI-1 at 6–12 h after traumatic injury. We analyzed the correlation between serum PAI-1 levels and other markers at admission and 6 h (Fig. 2a, b). Interestingly, PAI-1 levels at 6 h were most strongly associated with serum histone H3 levels at admission (rho = 0.68, *P* < 0.01) and 6 h (rho = 0.84, *P* < 0.01). Considering that extracellular histones can activate Toll-like receptors (TLRs) [25] and TLR activation may lead to PAI-1 expression [26, 27], a positive correlation between PAI-1 levels and histone H3 levels is reasonable.

### α2-PI levels are associated with blood transfusion

**Fig. 3:**
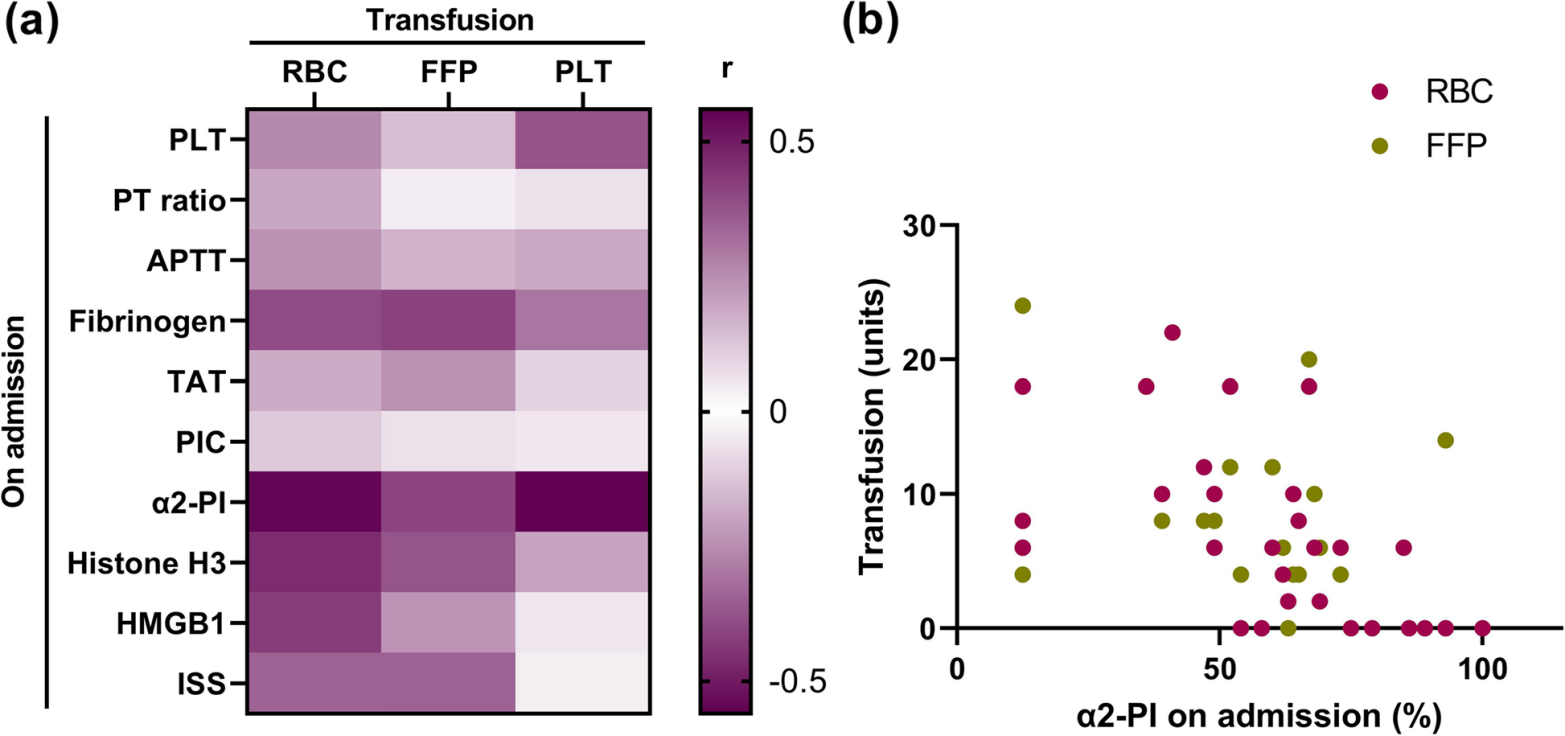
Alpha2-plasmin inhibitor levels are associated with the amount of blood transfusion. **a** Relationships between the amount of blood transfusion and platelet (PLT) counts, prothrombin time (PT), activated partial thromboplastin time (APTT), fibrinogen, thrombin-antithrombin complex (TAT), alpha2-plasmin inhibitor (α2-PI), plasmin-α2-PI complex (PIC), histone H3, high mobility group box 1 protein (HMGB1), and Injury Severity Score (ISS) levels in patients with severe trauma are shown. The intensities of the correlation are color-coded according to Pearson’s r values, with deep purple colors indicating strong correlations. **b** Correlation between α2-PI levels on admission and the amount of red blood cell (RBC) transfusion and fresh frozen plasma (FFP) transfusion are shown

Next, we examined the association between the amount of blood transfusion and coagulation and fibrinolysis markers and DAMPs (Fig. 3a, b). Among these markers, α2-PI levels on admission had the strongest negative correlation with the amount of red blood cell transfusion (Pearson’s *r* =  − 0.55, *P* < 0.01), fresh frozen plasma transfusion (*r* =  − 0.41, *P* < 0.05), and platelet transfusion (*r* =  − 0.56, *P* < 0.01). These findings are reasonable because decreased α2-PI levels can lead to dysregulated fibrinolysis, increased bleeding, and increased blood transfusion.

### Fibrinolysis perturbation and elevation of DAMP levels are associated with fatal outcomes

**Fig. 4:**
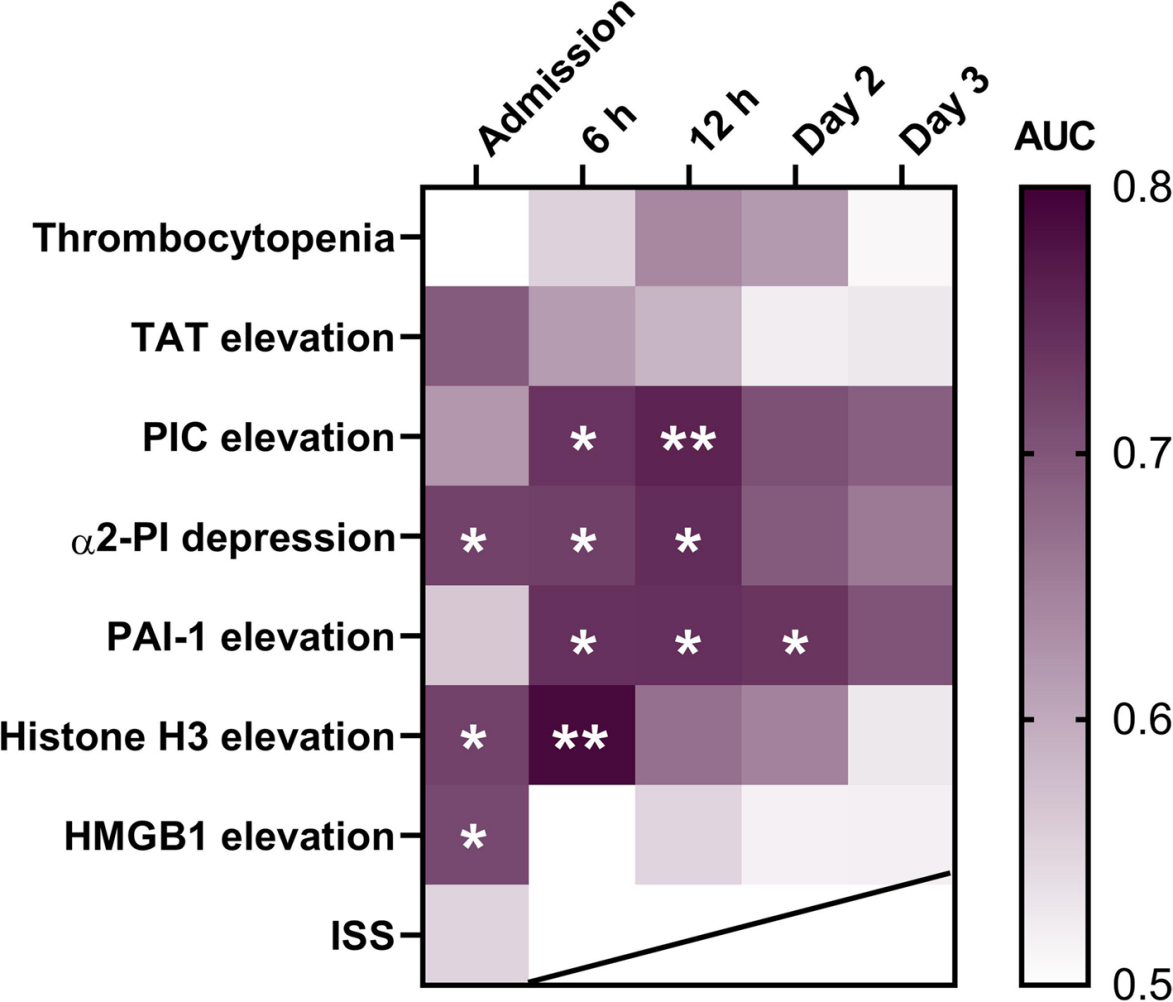
Fibrinolysis perturbation and elevation of DAMP levels are associated with fatal outcomes. Relationships between in-hospital death and thrombocytopenia, thrombin-antithrombin complex (TAT) elevation, alpha2-plasmin inhibitor (α2-PI) depression, plasmin-α2-PI complex (PIC) elevation, plasminogen activator inhibitor-1 (PAI-1) elevation, histone H3 elevation, high mobility group box 1 protein (HMGB1) elevation, and Injury Severity Score (ISS) levels in patients with severe trauma at admission, 6 h, 12 h, day 2, and day 3 are shown. The predictive performance for in-hospital death is color-coded according to the area under the curve (AUC) of the receiver operating characteristics curve, with deep purple colors indicating high predictive performance. *P < 0.05. **P < 0.01

We then searched for markers highly involved in fatal outcomes. As shown in Fig. 4, increased PIC levels at 6 h (AUC = 0.74, 95% confidence interval [CI]: 0.54–0.93) and 12 h (AUC = 0.76, 95% CI: 0.56–0.95) were associated with in-hospital mortality. Furthermore, increased histone H3 levels at admission (AUC = 0.72, 95% CI: 0.53–0.91) and at 6 h (AUC = 0.79, 95% CI: 0.62–0.96) were associated with fatal outcomes. Increased HMGB1 levels at admission (AUC = 0.71, 95% CI: 0.52–0.90) were also associated with fatal outcomes. In contrast to these fibrinolysis markers and DAMPs, coagulation markers had a limited association with fatal outcomes.

## Discussion

This study showed dynamic changes in coagulation markers, fibrinolysis markers, and DAMPs during the post-traumatic injury period. TAT, a marker of coagulation activation, was increased immediately after injury and then gradually decreased over time. PIC, a marker of fibrinolysis activation, was also elevated soon after the injury and then decreased over time. Furthermore, histone H3, a marker and mediator of cellular damage, reached a peak immediately after the traumatic injury. In contrast, PAI-1, an inhibitor of fibrinolysis systems, remained low at the time of admission, and then showed a marked increase at 6–12 h after traumatic injury.

TAT, an irreversible product of thrombin inhibition by antithrombin, is a biological marker of ongoing coagulation [28]. The present study observed TAT elevation soon after trauma. Although some studies [29, 30] suggested that traumatic coagulopathy early post-injury was not related to coagulation activation, others [31, 32] reported the excessive activation of coagulation in the acute phase of major trauma. Our data may support the latter idea, and suggest that consumption of coagulation factors also contribute to the development of TIC.

Recent studies indicated that plasma from trauma patients contained circulating procoagulant activity that spontaneously initiated coagulation throughout the vascular system, not just at the wound site [31, 33]. Furthermore, trauma patients had increased levels of plasma tissue factor antigen, which correlated with increased levels of thrombin generation *in vivo* [34]. Moreover, DAMPs trigger intravascular thrombus formation, possibly by inducing tissue factor expression on monocytes, elevating tissue factor procoagulant activity, and promoting platelet aggregation [17]. In the present study, we observed a marked increase in DAMPs, including histone H3 and HMGB1, immediately after traumatic injury. These findings suggest that DAMPs-induced intravascular coagulation may also contribute to the development of TIC.

Fibrinolysis abnormality is also important in the pathogenesis of TIC [5, 35]. Shock-induced tissue hypoxia/ischemia increased the release of t-PA from the endothelium, leading to the activation of fibrinolysis [34]. Previous studies showed that patients with TIC had high t-PA antigen levels, high plasmin generation, and increased fibrinolysis at the time of admission to the emergency department [9, 32, 34]. Subsequently, the activated endothelium induced the production of PAI-1 protein [36], although some studies reported a reduction in PAI-1 levels because of the proteolytic degradation of PAI-1 by activated protein C [9, 37]. Our data showed dynamic two-phase changes in the fibrinolysis system: increased fibrinolysis at the early stage and the inhibition of fibrinolysis at the later stage. This might be explained by the fact that tPA is constitutively produced and instantly released by the endothelium upon stimulation whereas PAI-1 produced after stimulation takes several hours to be released [32].

The importance of fibrinolysis in TIC has been suggested by clinical trials of tranexamic acid (TXA), a competitive inhibitor of plasminogen activation [38, 39]. They showed that survival benefit was most evident in those who received TXA within 3 h of injury [38, 39]. Thus, the early stage of TIC with increased fibrinolysis may be the optimal therapeutic target of TXA, and TXA may worsen outcomes in the later stages of TIC with fibrinolysis shutdown. In this study, 71% of patients received TXA in the prehospital setting or in the emergency room (Table 1). Death due to hemorrhagic shock was absent in this study.

The rapid assessment of the requirement of a massive blood transfusion for bleeding trauma patients is crucial, but remains a challenge [40]. Despite significant advances in the research fields of thrombosis and hemostasis, there is no test that can predict and identify clinically relevant coagulopathy [8]. The European guideline on the management of major bleeding and coagulopathy following trauma recommends that routine practice include the early and repeated monitoring of hemostasis, using combined traditional laboratory determination [PT, platelet counts, and fibrinogen level] and/or a point-of-care PT international normalized ratio and/or a viscoelastic method [41]. The PT and APTT are single-point indicators of clot potential, with clot formation occurring when only approximately 5% of all physiologically relevant thrombin is formed [42]. These tests provide little information on whole blood clot kinetics, elongation and retraction, or platelet–fibrin contributions [30]. Recently, viscoelastic point-of-care tests have been increasingly used for the diagnosis and management of trauma-associated bleeding [8]. These tests have the advantage of obtaining results rapidly; however, they may not be sensitive enough to detect fibrinolysis abnormalities associated with severe trauma [3]. In this study, we found that α2-PI levels on admission can be used to estimate needs for subsequent blood transfusion. Low α2-PI may contribute to massive plasmin generation, severe bleeding, and the need for transfusion [43]. Further studies are needed to clarify the mechanism of trauma-associated bleeding and to identify patients at risk of significant hemorrhage at an early stage.

This study had several limitations. First, this was an observational study conducted at a single center with a relatively small study population. Larger validation studies are needed to confirm our results. Second, the effects of TXA, fluid resuscitation, and other treatments were not investigated in this study. These factors may influence coagulation/fibrinolysis marker levels and transfusion.

## Conclusion

The elevation of DAMP levels and fibrinolysis perturbation were associated with fatal outcomes in patients with a traumatic injury. Patients with low α2-PI levels on admission tended to require blood transfusion.

## Data Availability

The datasets used and/or analyzed during the current study are available from the corresponding author upon reasonable request.

## Abbreviations

AUC: Area under the curve
DAMPs: Damage-associated molecular patterns
ELISA: Enzyme-linked immunosorbent assay
HMGB1: High mobility group box 1
ISS: Injury Severity Score
PAI-1: Plasminogen activator inhibitor-1
PIC: Plasmin-alpha2-plasmin inhibitor complex
TAT: Thrombin-antithrombin complex
TIC: Trauma-induced coagulopathy
TLRs: Toll-like receptors
tPA: Tissue-type plasminogen activator
TXA: Tranexamic acid
α2-PI: α2-Plasmin inhibitor
